# Supplementary material for: Content-rich biological network constructed by mining PubMed abstracts
Source: BMC Bioinformatics. 2004 Oct 8;5:147. doi: 10.1186/1471-2105-5-147 (PMC528731; doi:10.1186/1471-2105-5-147)
Supplement: Additional File 2 — The original results of the above study (non-essential files are deleted to keep the file size under the limit set by BMC bioinformatics). [file 1471-2105-5-147-S2.bz2 › chilibotAdditionalFile2/dip05/180ID9417082E618/html/PLAUR_FGR.html]

 


 **PLAUR** and **FGR** 
  
Found 1 abstracts in PubMed, retrieved 1.  
 

 What does Google say? 
 PDF only 
| .edu only 

---
